# Rapid and gentle volumetric imaging of host–pathogen interactions in salmon skin cells using projective oblique plane microscopy

**DOI:** 10.1038/s41598-025-33371-2

**Published:** 2025-12-22

**Authors:** Jon-Richard Sommernes, Dhivya Borra Thiyagarajan, Florian Ströhl

**Affiliations:** 1 Department of Physics and Technology, UiT The Arctic University of Norway, Tromsø, Norway; 2 The Norwegian College of Fishery Science, UiT The Arctic University of Norway, Tromsø, Norway

**Keywords:** Biological techniques, Microbiology

## Abstract

High-speed, low-phototoxicity volumetric imaging is essential to quantify host–pathogen interactions in marine animal cell models, such as internalization of bacteria by Atlantic salmon skin keratocytes (SKCs). We developed an oblique plane microscopy (OPM) platform with an integrated projective OPM (pOPM) mode that preserves standard sample mounting and supports ultra-fast, optically sectioned projections. Leveraging the sparsity of bacterial signal, two pOPM projections at distinct viewing angles allow 3D localization by triangulation, reducing acquisition time and light dose by up to approximately 150-fold relative to full volumetric stacks. The hybrid workflow combines pOPM for rapid, gentle detection with OPM for volumetric context. We characterized system performance using 120 nm fluorescent beads and achieved near-diffraction-limited resolution across a 20 μm depth. In live, two-colour imaging of infected primary SKCs, we acquired 238 × 158 × 18 µm^3^ volumes in 5.3 s and performed 3 h time-lapse recordings at 10 min intervals at 4 h, 28 h, and 52 h post-infection. Automated analysis segmented cells, detected bacterial clusters, and classified their spatial relationship to cells, revealing internalization fractions of approximately 18%, 54%, and 52% at 4 h, 28 h, and 52 h post-infection, respectively. These results demonstrate that OPM and pOPM can quantify SKC–bacteria interactions with subcellular resolution over extended fields and timescales, providing a platform to investigate mechanisms of bacterial internalization and clearance relevant to aquaculture health.

**Supplementary Information:**

The online version contains supplementary material available at 10.1038/s41598-025-33371-2.

## Introduction

**Fig. 1:**
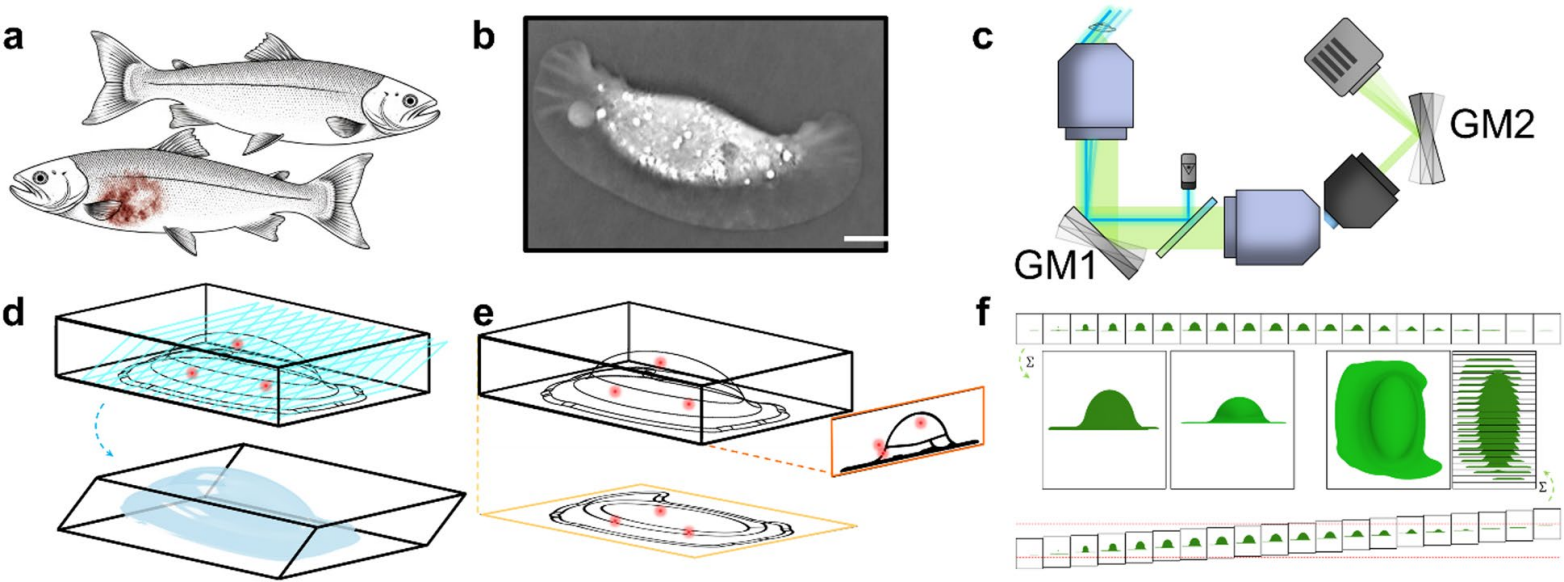
Concept and application overview. (**a**) Two salmon illustrating healthy skin versus winter ulcer lesions. (**b**) Example SKC holotomography image (Nanolive; adapted from prior work). (**c**) Simplified oblique plane microscopy (OPM) schematic with co-moving galvanometric mirrors (GM). (**d**) Volumetric OPM imaging of an infected SKC and representative membrane-labeled data. (**e**) pOPM projection imaging of bacteria with registration to the OPM cell volume. (**f**) Projection formation by sweeping the effective focal plane and imposing a synchronized shear. Scale bar is 10 μm.

Winter ulcers, frequently caused by Moritella viscosa (MV), are a persistent welfare and economic challenge in Atlantic salmon aquaculture, contributing to increased morbidity and mortality during colder seasons^1, 2^. Despite advances in vaccination, protection remains incomplete, and skin lesions continue to affect farmed salmonids^3^. The skin acts as a primary barrier and is populated by migratory skin keratocyte cells (shown in Fig. 1) that maintain barrier function, contribute to wound healing, and actively scavenge foreign material^4–9^. Prior work shows that epidermal keratocytes can ingest bacteria and discriminate between bacterial types, and more recent studies demonstrated uptake of micro- and nanoparticles by salmon skin and corneal epithelial cells^10, 11^. However, the timing and dynamics of bacterial uptake, interactions at the cell periphery, and subsequent intracellular processing remain incompletely understood.

Quantitative, real-time three-dimensional (3D) imaging of primary cells over extended fields of view (FoV) and time periods is critical to elucidate host–pathogen interactions under physiologically relevant conditions. Imaging methods must balance spatial and temporal resolution against phototoxicity, while accommodating conventional sample mounting and mechanical stability. Point-scanning confocal microscopy provides optical sectioning but is limited by sequential acquisition, photobleaching, and phototoxicity during long imaging sessions^12^. Spinning disk confocal systems parallelize pinholes and can achieve high frame rates but subject the entire sample to widefield illumination, constraining long-term viability^12, 13^. Light-sheet fluorescence microscopy (LSFM) reduces out-of-focus exposure and photodamage via selective plane illumination, but many implementations require nonstandard mounting geometries that complicate live-cell work with marine samples or are sensitive to refractive index mismatches introduced by saline media^14–25^.

Oblique plane microscopy (OPM), also known as swept confocally-aligned planar excitation (SCAPE) microscopy, adapts the principles of light-sheet imaging to a single-objective configuration^26–36^. In OPM, illumination and detection use the same high numerical aperture (NA) primary objective, enabling standard mounting while preserving optical sectioning and efficient light collection. A remote focusing system (RFS) re-images the oblique illuminated plane to an intermediate image^37–39^, which is then captured by a tilted detection arm to obtain 3D data within a rhomboidal volume without moving the sample. By placing a galvanometric mirror (GM) in a Fourier plane, the effective focal plane (EFP) can be swept rapidly through the sample for high-speed acquisition^27, 32^. OPM thus combines the photophysical advantages of light-sheet imaging with a straightforward sample geometry.

To further accelerate imaging and minimize dose, we integrated a projective OPM (pOPM) mode producing optically sectioned projections at selectable viewing angles in a single camera exposure as visualized in Fig. 1c-f^40–42^. pOPM sweeps the EFP through the specimen while imposing a synchronized shear on within the camera plane, equivalent to a shear-warp projection^43^. In sparse samples, such as bacteria in and around SKCs, two projections acquired at distinct angles localize emitters in 3D by triangulation without collecting a full volumetric stack, reducing acquisition time and light exposure by up to ~ 150× while maintaining comparable detail. Here we present a hybrid OPM + pOPM platform tailored to live imaging of SKC–MV interactions. We characterize system performance across a 20 μm depth window and demonstrate two-colour volumetric imaging of infected primary SKCs, capturing 238 × 158 × 18 µm3 volumes in 5.3 s and acquiring 3 h time-lapse series at 10 min intervals at 4 h, 28 h, and 52 h post-infection. Using an automated pipeline, we quantify bacterial internalization dynamics, revealing an increase in internalized fractions between 4 h and 28 h followed by a plateau at 52 h. These results establish OPM and pOPM as complementary tools for fast, gentle 3D imaging of host–pathogen interactions in marine cell systems.

## Results

### System performance characterization

**Fig. 2:**
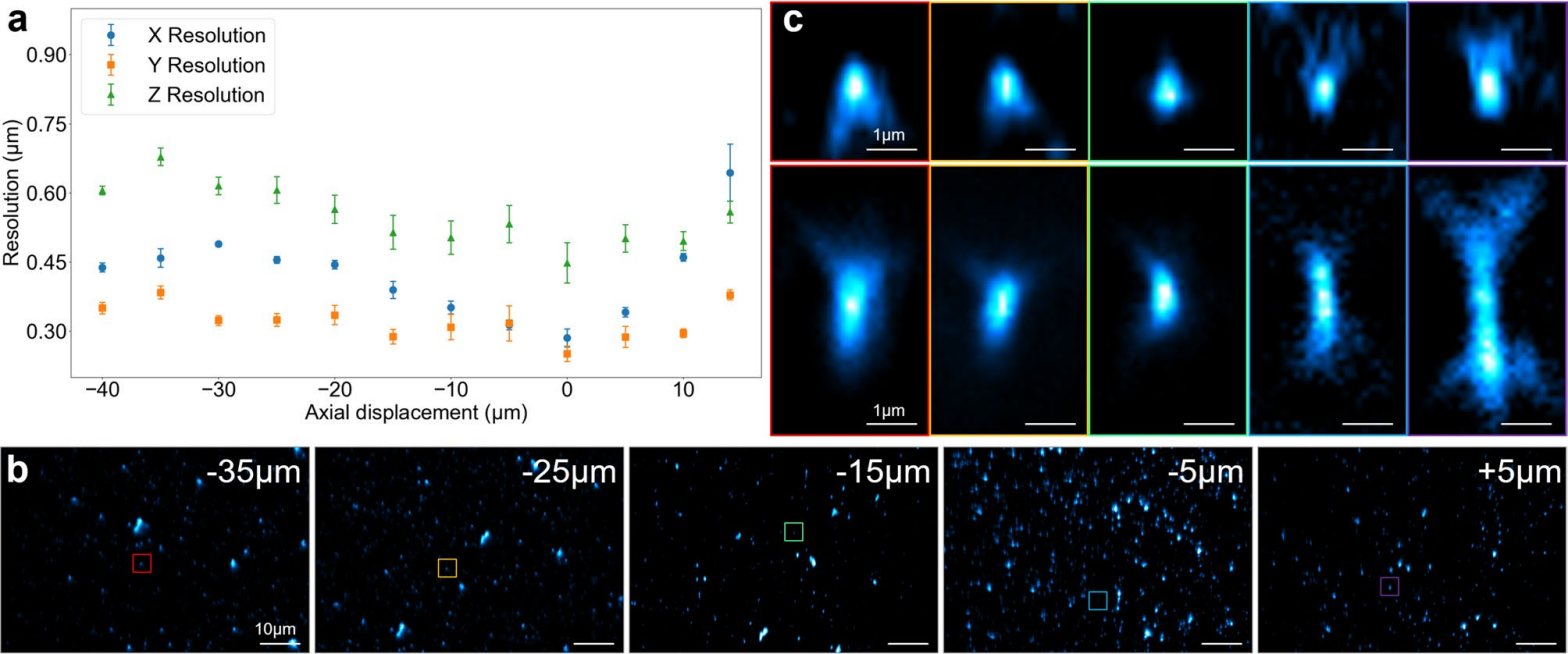
Resolution characterization with fluorescent beads. (**a**) Lateral and axial resolution across the remote image space from Gaussian fits to 120 nm beads. x denotes light-sheet scan direction; y is the orthogonal transverse axis; z is along the primary objective axis. (**b**) Representative bead images at selected displacements. A full 3D volume was acquired at each remote-space displacement. (**c**) Example XY and XZ zooms at the same axial displacements.

We assessed resolution using 120 nm fluorescent beads. At an emission wavelength of 626 nm, the theoretical diffraction limit under the employed conditions is approximately 232 nm laterally. We imaged a 2D bead sample at 5 μm steps in the remote image space; at each position we acquired a 3D stack and fit Gaussian profiles to estimate the point spread function (PSF) across the field. In the nominal focal plane, the measured full width at half maximum (FWHM) was 285 nm (x), 251 nm (y), and 448 nm (z), in close agreement with an OPM simulation (266 nm, 232 nm, and 445 nm, respectively)^44^, with deviations within 7% at the nominal plane. Resolution degraded gradually away from the nominal plane; we constrained the usable depth to maintain resolution within √2 of the nominal centre-resolution across all axes, yielding a practical depth of approximately 20 μm. For our samples, a 20 μm imaging depth provided a suitable balance of volumetric coverage and resolution (Fig. 2).

### Projective OPM enables rapid, optically sectioned projections

**Fig. 3:**
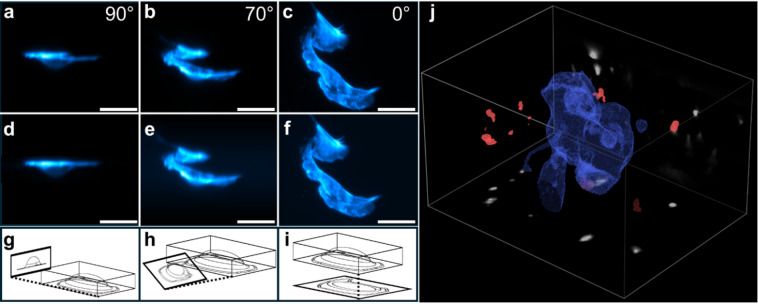
Optical versus computational projections and triangulation. (**a**–**c**) pOPM optical projections at orthogonal, oblique (~ 70°), and axial views. (**d**–**f**) Computational projections from OPM volumes at matched angles. (**g**–**i**) Schematic viewing directions. (**j**) Two-colour OPM volume of SKCs with bacteria, with two pOPM projections of the bacteria channel used for triangulation and registration. Sample: fixed SKC stained with Phalloidin–Atto 647 N. Scale bars: 30 μm.

We compared pOPM projections with computational projections from conventional OPM volumes. Volumetric datasets were acquired at 2 ms per plane (600 planes; volume time 3.6 s with rolling-shutter line delay). pOPM provided optical projections at selected viewing angles with a 50 ms exposure and a frame time of approximately 70.5 ms, yielding an approximately 50-fold reduction in acquisition time per projection relative to a full volume. Comparing full 3D localization precision (based on the data underlying Fig. 2) to a localization triangulation from projections, we find that the two approaches deliver results within 60 nm of each other. Intriguingly, pOPM projections preserved structural detail with less background haze than computational projections across multiple angles, including a lab-frame orthogonal view, an oblique view (~ 70°), and a view along the optical axis (Fig. 3a–i).

We validated the hybrid strategy by acquiring a two-colour OPM volume of SKCs with nearby bacteria, followed by two pOPM projections of the bacteria channel at distinct angles. Using a localization script, bacterial cluster positions were triangulated from the projections and registered to the OPM volume, showing good agreement with volumetric ground truth (Fig. 3j). Because only the in-focus plane contributes signal at any moment during pOPM exposure, projections benefit from optical sectioning and can be acquired at short exposure times with strong signal utilization. Brightest pixels used approximately 95% of the 16-bit well depth, indicating that exposure can be reduced further while maintaining adequate signal.

### Live two-colour OPM imaging of SKCs infected with *M. viscosa*

**Fig. 4:**
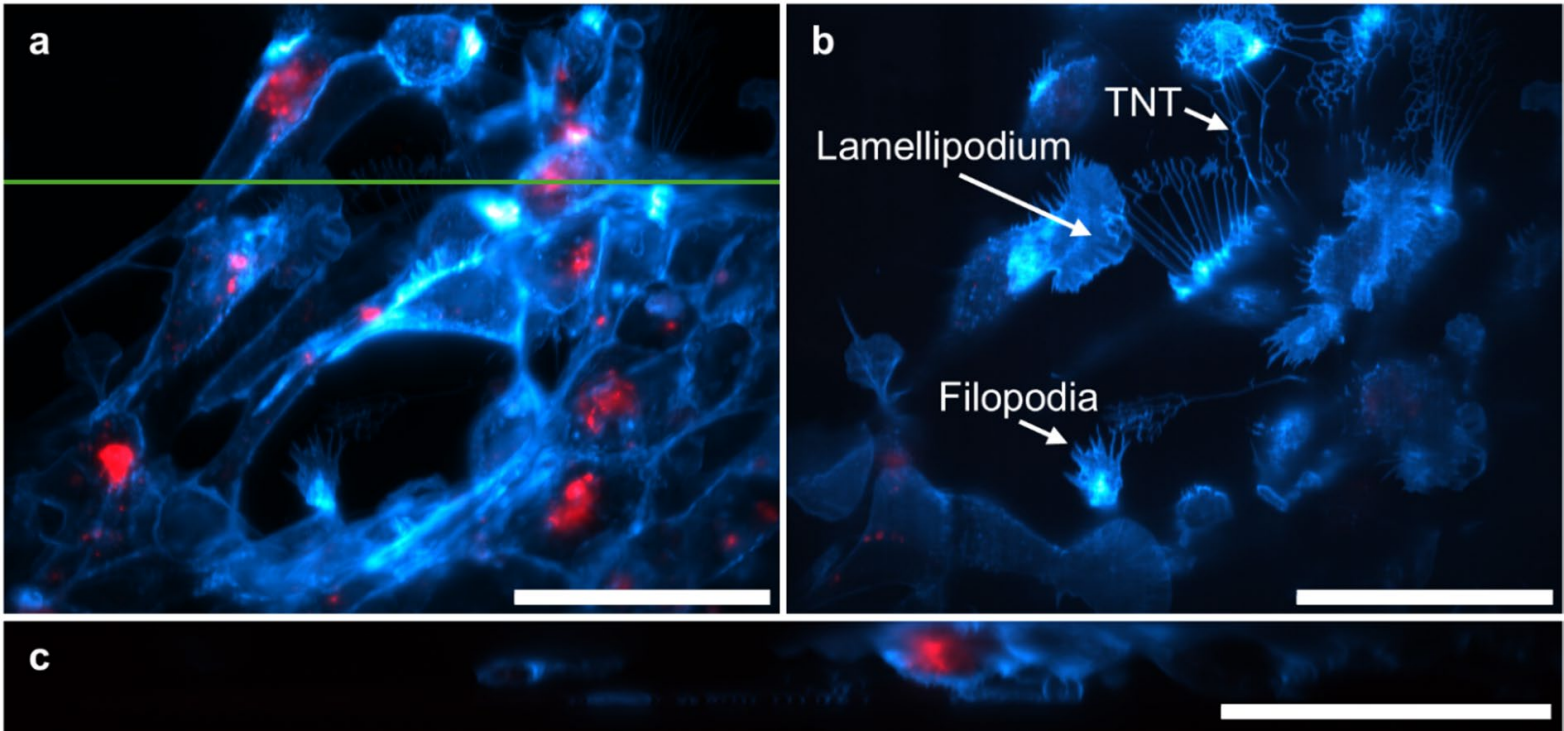
Live two-colour OPM imaging of infected SKCs. (**a**) Maximum intensity projection along z. (**b**) Single optical section at the glass interface resolving filopodia and lamellipodial features. (**c**) Side view at the position indicated in a. Cyan: SKCs (CellMask Green). Red: bacteria (BactoView Red). Scale bars: 50 μm.

For even faster recordings, we imaged primary SKCs exposed to MV using two-colour OPM with a dichroic splitter and two synchronized cameras, enabling simultaneous acquisition of cell (membrane-labelled) and bacteria channels without filter switching. For a 238 × 158 × 18 µm^3^ volume, the stack was acquired in 5.3 s at 10 ms per plane. We captured multiple cells within a single FoV and resolved subcellular features such as filopodia and lamellipodia with minimal out-of-focus background (Fig. 4). The frame rate and gentle illumination enabled long time series of fast-migrating keratocytes.

### Longitudinal quantification of bacterial internalization

**Fig. 5:**
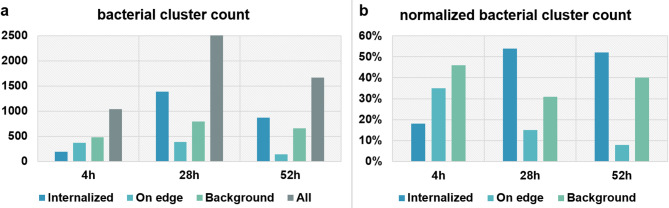
Quantification of bacterial internalization. (**a**) Counts of internalized bacterial clusters at 4 h, 28 h, and 52 h post-infection aggregated over nine FoVs per sample across 3 h time-lapse. (**b**) Normalized fractions of internalized bacterial clusters; classification performed by automated pipeline.

To quantify internalization dynamics, we acquired tiled time series comprising nine adjacent FoVs per sample at 10 min intervals over 3 h and imaged three independent samples at 4 h, 28 h, and 52 h post-infection. Three matched samples without bacteria served as references. An automated Python pipeline segmented cells, detected bacterial clusters, and classified each cluster as internalized, membrane-associated, or extracellular by overlap with the cell mask (see Methods and Supplementary Methods S1). At 4 h, the internalized fraction was approximately 18%. By 28 h, both the number of bacterial clusters and the internalized fraction increased, with approximately 54% classified as internalized. At 52 h, the total bacterial cluster count decreased relative to 28 h, and the internalized fraction remained at approximately 52% (Fig. 5). Bacterial clusters were frequently observed at cell edges, suggesting active interactions at the periphery prior to internalization. Formal statistical inference across biological replicates will be addressed in future studies.

### Imaging speed and dose considerations

Although pOPM can substantially reduce acquisition time and dose for sparse structures, conventional OPM with dual-camera usage provided sufficient performance for the present live-cell time lapses. Two-colour volumes were acquired at 10 ms per plane, and similar visualization quality was achieved at 6 ms per plane with strong signal and minimal background. Under lower bit depth (11-bit) and with additional optimization, such as leveraging axial aliasing to reduce the number of required planes in the scan direction, the volumetric rate could exceed 2 Hz for comparable volumes. We found that these optimizations were not necessary for SKC imaging in this study but may become relevant in recording internalization events as they occur.

## Discussion

Hybrid OPM + pOPM enables rapid, gentle, and quantitative 3D imaging of host–pathogen interactions in primary salmon skin keratocytes. By combining optically sectioned volumetric OPM with instantaneous, angle‑selectable pOPM projections, the platform supports long‑term, simultaneous two‑colour time‑lapse imaging and fast detection/localization of sparse bacterial signals with markedly reduced acquisition time and light exposure. This is well suited to saline marine media, where refractive‑index mismatches and saltwater compatibility can hinder dual‑objective light‑sheet systems. pOPM produces sectioned projections in a single exposure; for sparse objects, two projections at distinct angles suffice to triangulate 3D positions, reducing time and dose by up to ~ 150× compared to full stacks while preserving detail comparable to computational projections. In practice, pOPM provides a user‑friendly live “survey” mode for navigation and event detection, with immediate switching to OPM for volumetric acquisition when needed.

Using this platform, we observe a consistent trajectory in primary SKCs exposed to MV in which peripheral engagement is followed by increased internalization. The internalized fraction rises from approximately 18% at 4 h to about 50% by 28 h, with a sustained plateau thereafter (Fig. 5). The concurrent reduction in total bacterial counts by 52 h is compatible with a diminishing extracellular pool, potentially reflecting clearance, reduced adherence, or altered detectability under the labelling conditions used. The frequent presence of bacteria at cell edges is consistent with peripheral capture prior to uptake, although their distribution relative to specific subcellular structures was not quantified in this study. Taken together, these data support a model in which migratory epidermal keratocytes act as active sentinels that sequester bacteria from the skin surface. Before drawing definite conclusions, care must be taken as the used overlap-based internalization classifier can misassign tightly apposed bacteria at ruffled membranes, the 10 min sampling interval may miss short-lived entry or egress events, and imaging at ambient temperature following culture at 4 °C may introduce a thermal mismatch that could alter SKC and MV behaviours.

Nevertheless, the sustained high internalized fraction suggests that SKCs contribute to first-line defence by physically removing MV from the epithelial interface. Whether this reduces pathogen burden or, alternatively, provides an intracellular niche depends on the fate of internalized bacteria. Future studies incorporating endosomal/lysosomal markers, physiological temperature control, and event-trigger pOPM high-speed imaging should clarify uptake kinetics and intracellular processing, moving from descriptive sequestration dynamics toward mechanism and, ultimately, toward understanding how SKCs influence MV persistence or clearance in the salmon epidermis. More generally, our imaging approach is broadly applicable to sparse targets in complex cellular contexts (e.g., multi‑strain mixtures, immune cell–pathogen interactions). Simultaneous two‑colour acquisition reduces exposure versus sequential switching, and pOPM’s live projections speed screening and guide targeted volumetric imaging. Future technology-develop work includes higher‑speed volumetric OPM via exploiting axial aliasing, adaptive optics for saline environments, and more flexible temperature control.

## Methods

### Cells and bacteria

Primary skin keratocyte cells (SKCs) were isolated from Atlantic salmon (Salmo salar) scales as described previously^11^. Briefly, scales were removed from euthanized post-smolt fish using clean forceps and placed in culture or glass-bottom dishes. After 6–10 min, Hank’s Balanced Salt Solution (HBSS) supplemented with penicillin/streptomycin and amphotericin was added. After 2–3 days at 4 °C, cell sheets formed. For bacterial exposure, glycerol stocks of M. viscosa (kind gift from Nofima) were streaked on blood agar plates with 2% NaCl and incubated at 12 °C until colony formation (~ 48 h). Single colonies were cultured in FAMP medium to OD ~ 0.6–0.8, pelleted (10,000 rpm, 4 °C, 10 min), washed in 0.9% NaCl, and resuspended in HBSS. Bacteria were diluted to 10 − 2 ml − 1 for exposure. Cells were labeled with CellMask Green; live bacteria were labeled with BactoView Red. Full protocols are provided in Supplementary Methods S2.

### Microscopy and acquisition

Imaging was performed on a custom OPM platform with integrated pOPM. A high-NA primary objective (100× silicone immersion, Nikon) provided illumination and detection. A remote focusing system re-imaged the oblique illuminated plane, and a galvanometric mirror (GM1) in a Fourier plane swept the effective focal plane (EFP) through the sample for volumetric OPM. A second galvanometric mirror (GM2) in the detection arm imposed a synchronized shear during pOPM to generate optically sectioned projections at selectable viewing angles. Two-colour OPM used a dichroic splitter to direct channels to two synchronized sCMOS cameras.

Representative parameters: For live two-colour OPM, volumes of 238 × 158 × 18 µm3 were acquired in 5.3 s at 10 ms per plane. For pOPM, typical exposures were 50 ms per projection with a frame time of approximately 70.5 ms. For longitudinal studies, nine adjacent FoVs were imaged per sample at 10 min intervals over 3 h at 4 h, 28 h, and 52 h post-infection. The chosen intervals served to reach a clear change in the number of observed bacteria–cell interactions per sample, aiding a more robust descriptive statistics, while keeping data volumes within reasonable bounds. Complete system layout, component list, and per-dataset parameters are provided in Supplementary Methods S1.

### Image processing and analysis

OPM volumes were deskewed and rotated to the lab frame using an affine transform based on scan geometry. Two-colour volumes were co-registered via image-based registration using an image splitter configuration. For bacterial internalization quantification, a Python pipeline performed: (1) segmentation of the cell channel to generate a 3D binary cell mask; (2) detection of bacterial clusters by background thresholding of the bacteria channel; (3) 3D connected-component labeling with size filtering; and (4) overlap-based classification of each bacterial cluster as internalized, membrane-associated, or extracellular based on the fraction of voxels overlapping the cell mask. Full algorithmic details are provided in Supplementary Methods S1.

## Supplementary Information

Below is the link to the electronic supplementary material.

Supplementary Material 1

## Data Availability

Data supporting the findings of this study are as of now only available from the corresponding author upon reasonable request due to large file sizes, but raw and processed imaging datasets will be made available along with minimal metadata via a public repository in the future.

## Code availability

Custom analysis code (Python) for image deskewing, segmentation, bacterial detection, and overlap-based classification will be made available at a public repository [https://github.com/JRSommernes/OPM-pOPM-analysis]. The code will include documentation and example data sufficient to reproduce the main analyses.
