# Supplementary material for: Rapid and gentle volumetric imaging of host–pathogen interactions in salmon skin cells using projective oblique plane microscopy
Source: Sci Rep. 2025 Dec 22;15:45133. doi: 10.1038/s41598-025-33371-2 (PMC12748573; doi:10.1038/s41598-025-33371-2)
Supplement: Supplementary file 1 — Supplementary Material 1 [file 41598_2025_33371_MOESM1_ESM.pdf]

Supplementary Information for “Rapid and gentle volumetric imaging of host–pathogen interactions in salmon skin cells using projective oblique plane microscopy”

Jon-Richard Sommernes[1], Dhivya Borra Thiyagarajan[2], Florian Ströhl[1*]

1 Department of Physics and Technology, UiT The Arctic University of Norway, Tromsø, Norway

2 The Norwegian College of Fishery Science, UiT The Arctic University of Norway, Tromsø, Norway

Correspondence: florian.strohl@uit.no

Contents

- Supplementary Methods
  S1. System design and operation
  S2. Sample preparation and labelling
- Supplementary Figures S1–S3
- Supplementary References

Supplementary Methods

S1. System design and operation

S1.1 Optical layout and components

This platform integrates oblique plane microscopy [1–3] (OPM) and projective OPM [4,5] (pOPM) in a single optical path as shown in Figure S1. Illumination and detection share the same high numerical aperture (NA) primary objective (O1), enabling standard inverted-microscope mounting. A remote focusing system [6] (RFS) reconstructs the oblique illuminated plane at a remote image space, which is imaged by an angled tertiary objective (O3). The effective focal plane (EFP) is swept through the sample by a galvanometric mirror (GM1) placed in a Fourier plane. For pOPM, a second galvanometric mirror (GM2) imposes a synchronized shear on the intermediate image during a single camera exposure, producing an optically sectioned projection at a selectable viewing angle. A comprehensive part list, with reference to Figure S1, is as follows:

Reference	Part name	Use

O1	Nikon CFI SR HP Plan Apo Lambda S 100XC Sil	Primary objective
O2	Nikon CFI Plan Apochromat Lambda D 40X	Secondary objective
O3	ASI AMS-AGY v1	Tertiary objective
TL1-TL4	Thorlabs TTL200-A	Tube lens
SL1	Thorlabs CLS-SL	Scan lens
SL2	Thorlabs LSM03-VIS	Scan lens
M5	Thorlabs BBSQ2-E02	Mirror
M6	Thorlabs BB111-E02	Mirror
GM1/GM2	Thorlabs GVS211/M	Scanning mirror
DM2	AHF Quad Line Beamsplitter R405/488/561/635 lambda/5	Dichroic mirror
FW1/FW2	CAIRN OptoSpin25	Filter wheel
F1/F2/F3/F4	AHF F37-446, AHF F37-521, AHF F39-613, AHF F76-649	Filters
Camera1/Camers2	Teledyne Prime BSI Express	Camera
Laser 1	FISBA READYBeam™	Laser
Laser 2	COHERENT OBIS LS 561 nm	Laser
Col1	Nikon CFI Plan Fluor 4X	Collimating lens 1
Col2	Thorlabs AC254-100-A	Collimating lens 2
M1-M3	Thorlabs BBE1-E02	Mirror
DM1	AHF Laser-Shortpass-Beamsplitter zt 561 sprdc flat	Dichroic mirror
AS	Thorlabs VA100c/M	Adjustable slit
CL	Thorlabs ACY254-200-A	Cylindrical lens
DL	Thorlabs AC254-200-A	Lens
M4	Thorlabs CCM1-E02/M	Mirror
	National instruments PXIe-6738	Hardware controller
	Arduino UNO	Camera synchronizer

Henceforth, components will be referred to as the reference as shown in the left column of the part list.

S1.2 OPM operation

In this system, a sample is placed on a stage like a conventional inverted microscope. The system utilizes two lasers (Laser 1 and Laser 2) that are combined into a common path using a dichroic mirror (DM1). The beams are passed through a slit and a cylindrical lens (CL) and a doublet lens (DL) to create a light-sheet. The light-sheet is passed into the imaging path using a dichroic mirror (DM2). The light-sheet is then passed through the imaging system and into the sample.

The sample is imaged using a high-NA primary objective lens (O1). The back focal plane (BFP) of O1 is conjugated onto the surface of a GM (GM1) using a tube lens (TL1) and a scan lens (SL1). The mirror surface is then conjugated onto the BFP of the secondary objective (O2) using a scan lens (SL2) and a tube lens (TL3). After O2, the light passes through a tertiary objective (O3). The light then passes through an imaging element (IE), which is a swappable optical element in a cage cube. For this experiment, the IE used was a dichroic mirror (AHF F38-560_T3). Each beam passes through a filter wheel (FW1/FW2) containing identical emission filters (F1/F2/F3/F4) and a tube lens (TL4/TL5). In one beam path the light is passed over a GM (GM2), then both beams are imaged using a camera (Camera1/Camers2). The system is controlled using a NI DAQ board, and the cameras are synchronized using a microcontroller (Arduino UNO).

The optical path from O1 to O2 creates a remote focus system [6]. This system aims to create a stigmatic reconstruction of the sample volume at a remote image space, allowing the sample to be re-imaged by O3. As the image is a stigmatic reconstruction of the sample space, we can therefore re-image this volume at an angle. This allows us to shoot the light-sheet into the sample from the edge of the primary objective, creating a light-sheet coplanar with the effective focal plane (EFP) of the system.

As the system contains a RFS, this also allows the system to benefit from focal plane scanning using a GM [3]. By conjugating the GM onto the BFP of both O1 and O2, rotating the GM will induce a linear shift in the EFP in sample space. By passing the illumination and emission light over the same GM, the EFP and illumination plane will remain coplanar throughout the imaging sequence. While imaging, the emission will pass over the same GM, de-scanning the EFP by the same amount, keeping the EFP static in the remote image space. This allows the entire volume to be imaged by rotating a single GM without any additional moving parts.

As the system utilized light-sheet illumination, the segmentation and contrast of images are increased. This allows the system to image at lower laser power while keeping good signal to noise ratios, making the system minimally phototoxic. This low phototoxicity in addition to the fast scanning allowed by GM scanning makes the system capable of fast imaging over extended time series.

S1.3 pOPM operation

The operation principle of pOPM (main optical path shown in the secondary imaging arm in Figure S1) is depicted in Figure S2. During a single image acquisition, the first GM scans the EFP through the sample, while the second GM simultaneously scans the image on the sensor to impose the required shear. Because only the in-focus plane contributes signal at any instant, the integrated signal forms an optically sectioned projection of the entire volume. By adjusting the shear magnitude, pOPM yields projections at arbitrary angles about one rotation axis within a single camera exposure.

Because pOPM integrates fluorescence from the entire volume within a single exposure, it can operate with very short exposure times while still capturing information from the full sample. Unlike conventional OPM, where the EFP is oblique and requires post-processing to view in the lab frame, pOPM directly produces projections at arbitrary viewing angles, including the lab-frame, without any reconstruction. This enables a true live view of the whole specimen for navigation and monitoring. Running at high frame rates over the full FoV, pOPM is well suited for event detection where the system can watch for biological events and then switch to OPM for volumetric acquisition. Consequently, pOPM is a valuable addition for both user-friendly sample survey and rapid, gentle imaging when full 3D stacks are not required.

The system also supports efficient 3D localization in sparse specimens. In bacterial imaging, where only small regions contain signal within an otherwise empty FoV, two pOPM projections acquired at distinct angles are sufficient to triangulate bacterial positions in 3D without acquiring a full OPM volume. This approach substantially reduces acquisition time, light dose, and data volume, and can run at higher temporal resolution than conventional volumetric imaging. This can thus be used in combination with OPM to acquire images of cells and bacteria using a single camera, lowering the combined acquisition time and data volume significantly.

S1.4 pOPM triangulation for sparse 3D samples

For sparse, point-like signals (e.g., individual bacteria), we estimate 3D positions from two pOPM projection images taken at different shear settings, avoiding a full 3D stack. Conceptually, both projections share the same vertical axis in the image, while the horizontal position shifts with the viewing angle. The script uses a simple, orthographic model to assign coordinates: X is taken from the horizontal position in projection 1, Z from the horizontal position in projection 2, and Y from the shared vertical position (by default the average of the two). This provides fast 3D localization with much lower light dose, acquisition time, and data volume than conventional volumetric imaging, aligning with the pOPM goal of rapid survey in largely empty fields of view.

To prepare the data, the script loads the two projection images (TIFF), converts them to 8-bit if needed, and orients them so their vertical axes match. It then highlights small bright spots on a dark background by enhancing local contrast around features of the expected size, applies a light denoising step, and uses a robust, data-driven

threshold (based on the image median and variability) to separate spots from background. A morphological operation connects faint pixels into coherent blobs. From the resulting binary image, connected components are filtered by size to suppress noise, and the center of each blob is measured to yield a set of 2D point coordinates for each projection.

The script matches the same spot across the two projections by comparing vertical positions only, which is both fast and reliable under the pOPM geometry. Vertical coordinates are first normalized by image height to handle small size differences between the two images. Spots are paired if their vertical positions agree within a user-defined tolerance (specified in pixels or as a fraction of image height). When multiple pairings are possible, a global matching step (Hungarian/optimal assignment, with a greedy fallback) selects the overall best set of pairs. The code provides an overlay visualization to inspect matched and unmatched detections for quality control. Finally, 3D coordinates are computed directly from the matched pixel positions as described above. Two output options are available: a straightforward "absolute-pixel" mapping, and a configurable variant that allows per-axis centering, scaling, and axis flips to align with a chosen world frame or physical units. Results (matches and 3D points) are saved in standard CSV/NPZ formats. The method works best when emitters are sparse and well separated along the vertical direction; ambiguous cases can be mitigated by tightening the matching tolerance and adjusting spot-size and area thresholds.

S1.5 Image analysis

After OPM acquisition, the volume needs to be processed before being analyzed. Although the EFP is swept through the sample at an oblique angle as shown in Figure S3a, the camera saves the frames as a conventional z-stack. Therefore, we apply an affine transformation to shear and rotate the image planes to align the data to the lab frame. The top row of Figure S3c shows the unsheared raw stack; The bottom row of Figure S3c shows the corrected volume after affine transformation, which accurately represents the sample geometry.

Using the OPM imaging mode, we acquired long time-lapse volumes to quantify bacterial internalization. To analyze internalization, we implemented a Python pipeline that classifies each bacterium based on its spatial relationship to the cell mask. The workflow comprises four steps:

1. Cell segmentation: The cell channel is segmented to produce a binary mask of cells against background, yielding a clean cell-volume mask.
2. Bacteria detection: The bacteria channel is background-thresholded to generate a binary mask of bacteria.
3. Connected components: A 3D connectivity analysis is applied to the bacteria mask to label individual bacteria or clusters. Objects below a size threshold are removed to suppress noise, leaving only plausible bacterial objects.
4. Overlap-based classification: For each labeled bacterium B, we compute the overlap fraction $f = |B \cap C| / |B|$, where C is the cell mask and $|\cdot|$ denotes voxel count. Classification rules are:
    a. Internalized: $f \geq 0.98$

b. Membrane-associated (edge): $0.05 \leq f < 0.98$
c. Extracellular (background): $f < 0.05$

The pipeline was applied to every FoV at each time point. For each sample, nine preselected FoVs were imaged at 10-minute intervals over a 3-hour period, yielding 20 time points per FoV (180 volumes per sample). This protocol was repeated for three independently prepared samples imaged at 4 h, 28 h, and 52 h post-infection, respectively, providing three 3-hour time-lapse series spaced approximately 24 hours apart.

To approximate native conditions prior to imaging, samples were stored at 4°C and then transferred to the microscope. Imaging was conducted at a stable 21°C due to the absence of stage/objective cooling and because the immersion primary objective is not compatible with low-temperature operation. While this temperature shift is a limitation that may influence cellular dynamics, the temperature was held constant during each time-lapse to ensure internal consistency across FoVs and time points.

S2. Sample preparation and labelling

S2.1 Cell Isolation

Keratocytes from scales were obtained in the lab like previous studies5. Briefly, for skin keratocytes, individual scales were pulled from skin using clean forceps and placed in either cell culture dishes or glass bottom dishes according to the requirement. The scales were left in the dish for 6-10 minutes before adding HBSS mix (10ml of 1000U penicillin/100μg streptomycin mix and 4ml 100μg of amphotericin). About 2-3 days after scale harvesting and seeding (4◦C) cell sheets of avalanches were formed.

S2.2 Bacterial culture and infection

Glycerol stocks of *M. viscosa*, were obtained from Nofima as a kind gift. These have been described before. Glycerol stock were streaked onto blood agar plates with 2%NaCl (provided by National Veterinary Institute, Ås, Norway) and incubated at 12◦C until colony formation. At approximately 48 hours colonies were formed from all the strains; these colonies were tested to confirm the presence of respective bacterial species using the Mono aqua-test system (Bionor laboratories AS). After confirmation of each strain, single colonies were picked and grown in liquid media. MV were grown in FAMP media (5g tryptone, 15g Marine broth, 700ml Milli-Q water, and 300ml saltwater). Once OD reached OD ~0.6-0.8, bacterial cells were harvested by centrifuging at 10000 rpm at 4◦C for 10 minutes. Cell pellets were washed using 0.9% NaCl before the bacteria were suspended in HBSS. After washing, the bacterial pellets were re-suspended and diluted to 10^{-2}/ml and this dilution which was used to expose the keratocyte cells. Cell Mask green (C37608) were purchased from ThermoFisher Scientific (USA). BactoView live fluorescent bacteria stains from Biotium (40102-T) was used to stain live bacteria for visualization of bacterial internalization in keratocytes.

Supplementary Figures

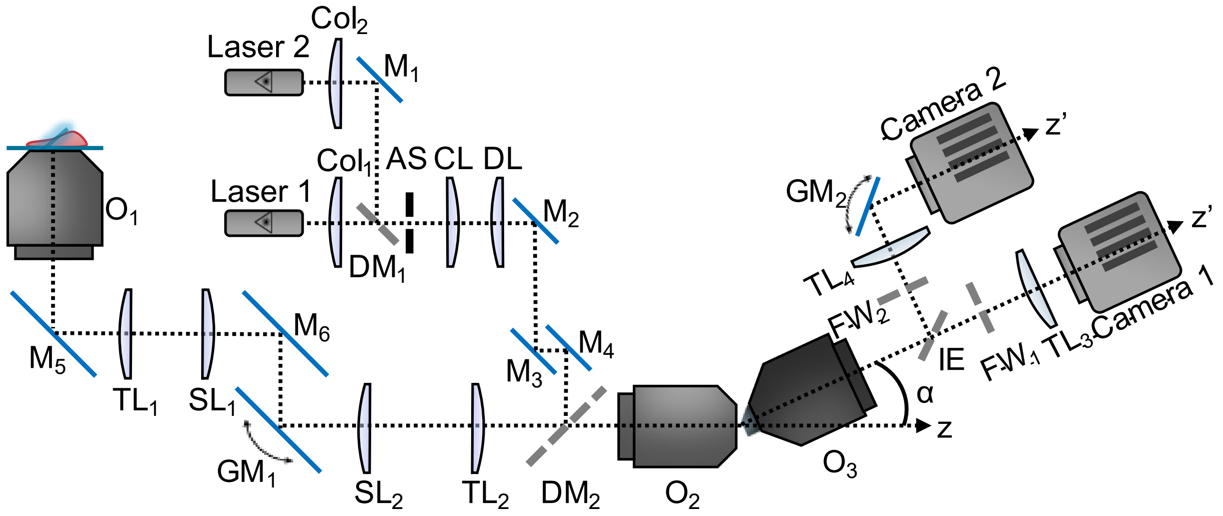

Supplementary Figure S1. Detailed optical schematic of the OPM+pOPM instrument. Full optical diagram showing lasers, beam combination (DM1), light-sheet formation (slit, CL, DL), injection dichroic (DM2), primary objective (O1), Fourier-plane relay (TL1, SL1, GM1, SL2, TL3), secondary objective (O2), tertiary objective (O3), imaging element IE splitting into two detection arms (FW1/FW2, TL4/TL5, cameras), and the projective shear mirror (GM2) in one detection arm. The tilt angle α between O2 and O3 was set to 25°.

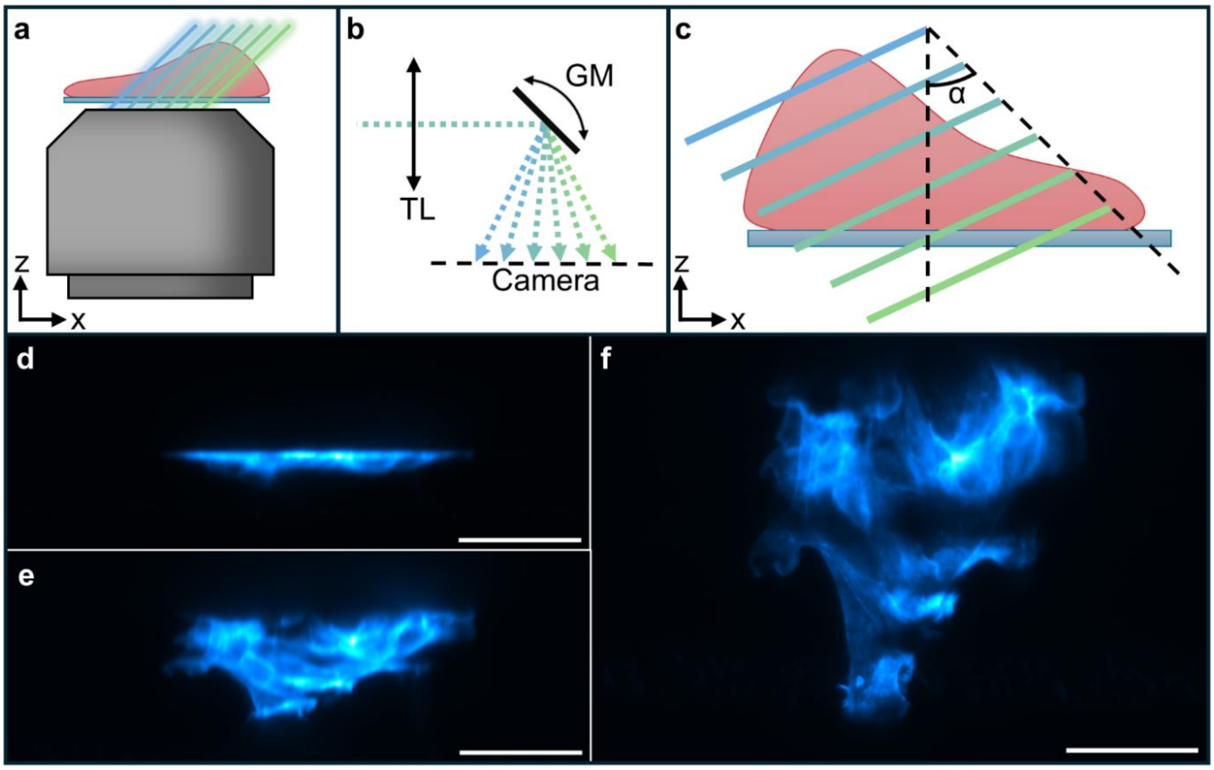

Supplementary Figure S2. Depiction of pOPM imaging. Panel a show how the focal plane scans through the sample from the starting position (green plane) to the end position (blue plane). Panel b shows how the light goes through the last tube lens

and is scanned onto the camera by the second GM, inducing a shear in the volume between the optical axis at the start of the acquisition (green line) and the end of the acquisition (blue line). Panel c shows how this shear in image volume shifts the focal plane in the sample space from the start of the acquisition (green line) to the end of the acquisition (blue line). In panels a-c, the scan is shown by a series of lines of gradually shifting color, indicating how the scan is performed. Panels d-f show projection images at 90°, 65°, and 0° respectively. The projection images are of SKCs, stained with Phalloidin–Atto 647N. All scale bars are 50μm.

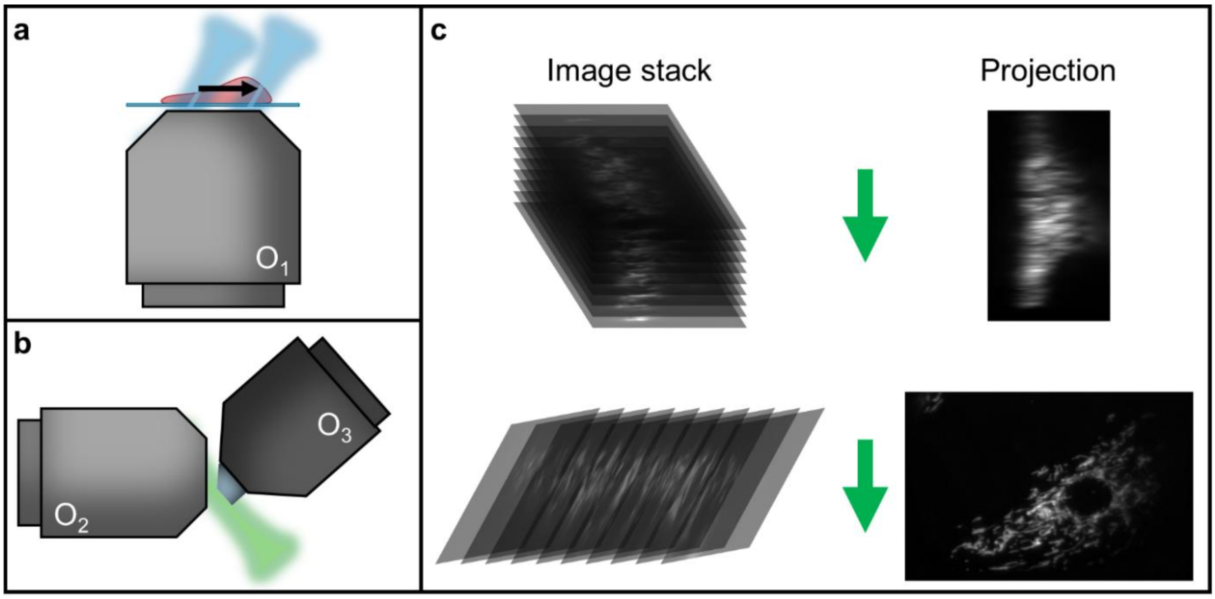

Supplementary Figure S3. Figure showing the acquisition process of the OPM system. Panel a show how the light-sheet, and equivalently the effective focal plane, is translated through the sample. During a full volume acquisition, the light-sheet is translated linearly through the sample, as shown by the two blue beam waists and the black arrow showing the translation direction. Panel b shows how the focal plane is reconstructed in the remote image plane. Due to the de-scanning effect of the GM, the effective focal plane will always be at a static plane in the remote space, being able to be re-imaged by O3. Panel c shows how the data is acquired by the acquisition engine and reconstructed by an affine transformation. The top row shows how the data is stored during an acquisition, while the bottom row shows the data after an affine transformation. The stacks in each row are projected along the axis indicated by the green arrow, resulting in the projection shown to the right of the stacks.

Supplementary References

1. C. Dunsby, "Optically sectioned imaging by oblique plane microscopy," (2008).
2. M. B. Bouchard, V. Voleti, C. S. Mendes, C. Lacefield, W. B. Grueber, R. S. Mann, R. M. Bruno, and E. M. C. Hillman, "Swept confocally-aligned planar excitation (SCAPE) microscopy for high-speed volumetric imaging of behaving organisms," Nature Photon **9**, 113–119 (2015).

3. M. Kumar, S. Kishore, J. Nasenbeny, D. L. McLean, and Y. Kozorovitskiy, "Integrated one- and two-photon scanned oblique plane illumination (SOPi) microscopy for rapid volumetric imaging," Opt. Express **26**, 13027 (2018).
4. B.-J. Chang, J. D. Manton, E. Sapoznik, T. Pohlkamp, T. S. Terrones, E. S. Welf, V. S. Murali, P. Roudot, K. Hake, L. Whitehead, A. G. York, K. M. Dean, and R. Fiolka, "Real-time multi-angle projection imaging of biological dynamics," Nat Methods **18**, 829–834 (2021).
5. B. Chen, B.-J. Chang, S. Daetwyler, F. Zhou, S. Sharma, D. M. Lee, A. Nayak, J. Noh, K. Dubrovinski, E. H. Chen, M. Glotzer, and R. Fiolka, "Projective light-sheet microscopy with flexible parameter selection," Nat Commun **15**, 2755 (2024).
6. E. J. Botcherby, R. Juškaitis, M. J. Booth, and T. Wilson, "An optical technique for remote focusing in microscopy," Optics Communications **281**, 880–887 (2008).
